# Immunomodulatory Effects of Green Tea Catechins and Their Ring Fission Metabolites in a Tumor Microenvironment Perspective

**DOI:** 10.3390/molecules29194575

**Published:** 2024-09-26

**Authors:** Emmanuele D. S. Andrade, Ronimara A. Santos, Landi V. C. Guillermo, Noriyuki Miyoshi, Danielly C. Ferraz da Costa

**Affiliations:** 1 Graduate School of Integrated Pharmaceutical and Nutritional Sciences, University of Shizuoka, Shizuoka 422-8526, Japan; manu.dutra.pbi@gmail.com; 2 Laboratory of Pathophysiology and Biochemistry of Nutrition, Department of Basic and Experimental Nutrition, Institute of Nutrition, Rio de Janeiro State University/UERJ, Rio de Janeiro 20550-013, Brazil; ronimaras@gmail.com; 3 Laboratory of Investigation on Mechanisms of Immunoregulation, Department of Microbiology and Parasitology, Biomedical Institute, Federal State University of Rio de Janeiro/UNIRIO, Rio de Janeiro 22290-240, Brazil; landiguillermo@gmail.com

**Keywords:** green tea catechins, catechin metabolites, immune response, tumor microenvironment

## Abstract

Green tea is the second most consumed beverage following water, and the health benefits provided by its consumption have been well established from research in recent decades. The main bioactive compounds found in all *Camellia sinensis*-based teas are catechins, which have been reported to have antioxidant, anticancer, anti-inflammatory, and immunomodulatory properties. Although most of the health benefits are well established, studies show that the intact catechins as found in tea are poorly absorbed in the digestive tract. These compounds are degraded and undergo ring fission by the gut microbiota, increasing their absorption. In this review, we gather knowledge of the health benefits of green tea catechins and their metabolites, with a particular emphasis on the immunomodulatory effects in a cancer microenvironment scenario.

## 1. Introduction

Green tea, a product of *Camellia sinensis* leaves, is one of the most widely consumed non-alcoholic beverages in the world. Depending on the type of processing the plant is subjected to, different teas can be obtained, such as white, green, yellow, oolong (also known as red tea), black, and dark tea (Pu-erh) [1]. Among all the *Camellia*-based teas, green tea is the most popular; thus, its health benefits have been extensively studied in recent decades [1,2].

Green tea exhibits antioxidant, anti-tumoral, anti-inflammatory, and immunomodulatory properties [1,2]. These benefits are primarily attributed to catechins, which are the main group of polyphenols in tea and a distinct class of flavonoids (flavan-3-ol). The key catechins in green tea include (−)-epicatechin (EC), (−)-epicatechin-3-gallate (ECG), (−)-epigallocatechin (EGC), and the most abundant one, (−)-epigallocatechin-3-gallate (EGCG) [3]. Although several studies have established the benefits of green tea, many have focused on the isolated catechin (EGCG), which is poorly absorbed in the intestinal tract of humans and rats. Catechins undergo degradation and ring fission by the intestine microbiota, which enhances their absorption [4,5,6]. Following digestion, eleven different metabolites have been identified from EGCG, with 5(3,5-dihydroxiphenyl)γ-valerolactone being the predominant metabolite [4,7].

This review aims to summarize current knowledge on the immunomodulatory effects of catechins and the main green tea metabolite, with a particular perspective on the tumor microenvironment and the consequent implications for cancer prevention.

## 2. Green Tea Consumption

Green tea stands out as the most popular among all *Camellia*-based teas. It is obtained through stages including withering, fixation (by panning or steaming), rolling or forming, and drying of the plant leaves [1]. Factors such as geographic region and climatic conditions at the time of cultivation can impact its chemical composition, as well as the degree of leaf ripeness, the time of year of harvest, and the conditions of processing and storage [8]. Altitude, temperature, humidity, rainfall, the pH and mineral content of the soil, and sunshine hours are some region and climatic characteristics which can affect tea quality and chemical composition [8,9,10]. Catechins are susceptible to degradation because of their reactive nature, being affected by heat and light exposure. Therefore, proper storage conditions and correct packaging are important to maintain tea quality [8,11].

**Figure 1:**
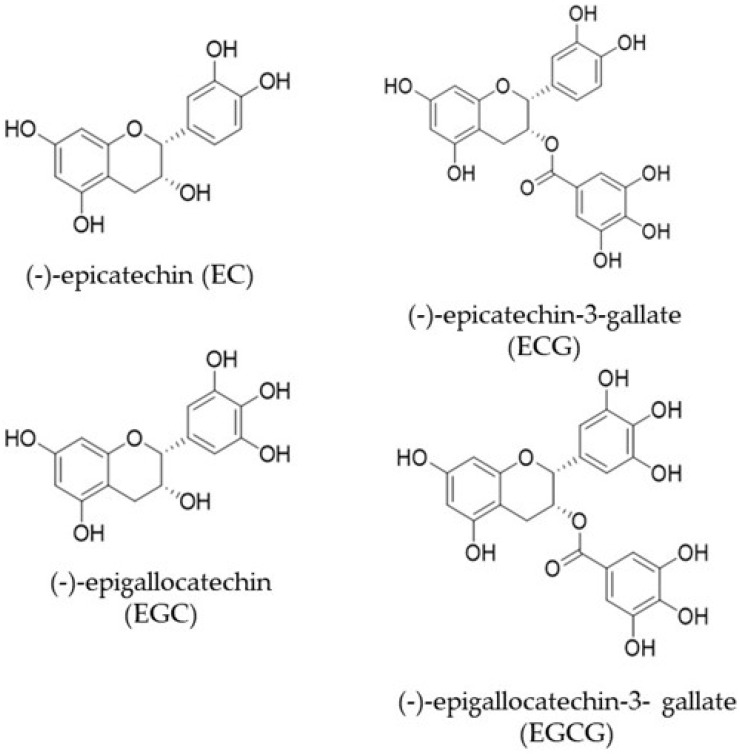
The main catechins in green tea.

Despite variations in its composition, phenolic compounds predominate, since green tea does not undergo the fermentation stage, which is known to decrease the concentrations of these bioactive compounds. The steaming step in green tea processing causes the inactivation of the oxidative enzyme polyphenol oxidase, which prevents the oxidation of most polyphenolic compounds (catechins, flavones, anthocyanins, phenolic acids, and tannins) [1,8]. Catechins stand out among them, accounting for 30 to 42% of the dry extract of the plant, the main ones being EC, ECG, EGC, and EGCG (Figure 1). EGCG has been established as the most abundant catechin, accounting for 50–80% of the total catechins in the dry extract [1,12]. Catechins are colorless, water-soluble compounds, and their concentration in the beverage is responsible for the astringency and bitterness of teas [12].

## 3. Bioavailability of Catechins

Once ingested, the phenolic compounds in green tea are only partially absorbed in the upper small intestine [13]. Studies using rats showed that less than 5% of catechins from an orally administered dose of tea reach the bloodstream [5,14]. Pre-clinical studies indicate an inverse relationship between the molecular mass of catechins and their bioavailability. The maximal plasma levels of EGCG (458 M.W.) after taking 200 mg of isolated EGCG are in the range of 0.26 μM, despite green tea containing 60–80% more EGCG than other catechins. Conversely, after consuming the same quantity of lower molecular weight catechins, higher plasma levels of EC (290 M.W.) and EGC (306 M.W.) were observed at 0.48 and 0.19 μM, respectively [15].

In humans, the highest levels of catechin plasma concentrations appeared to occur 1–2.5 h after intake. EGCG has a longer half-life (~5 h) than EGC and EC (~3 h), contrasting with its limited bioavailability [16]. Catechins’ biotransformation occurs immediately in phase II of metabolism, since the structure of phenolic compounds makes them unfavorable substrates for the enzymes of the cytochrome P450 complex [17]. The type of biotransformation a catechin undergoes affects its absorption, distribution, metabolism, excretion, and toxicity [8,18]. Additionally, studies on the metabolism of green tea catechins have been carried out in vitro, using human liver microsomes, human placental cytosol, human jejunal cytosol, and human saliva [19,20]. In human liver microsomes, EGC and EGCG were shown to undergo methylation and glucuronidation [21]. In human jejunal cytosol, (−)-epicatechin was shown to undergo sulfation, while in human saliva, EGCG was hydrolyzed into EGC and gallic acid [22,23]. In vitro studies using human placental samples showed that (−)-epicatechin, (+)-epicatechin, and (−)-epigallocatechin were good substrates for metabolic O-methylation by cytosolic catechol-O-methyltransferase (COMT), having a metabolism rate of 150–500 pmol/mg of protein/min, while (−)-epicatechin gallate and (−)-epigallocatechin gallate were *O*-methylated at much lower rates (<50 pmol/mg of protein/min) [24].

**Figure 2:**
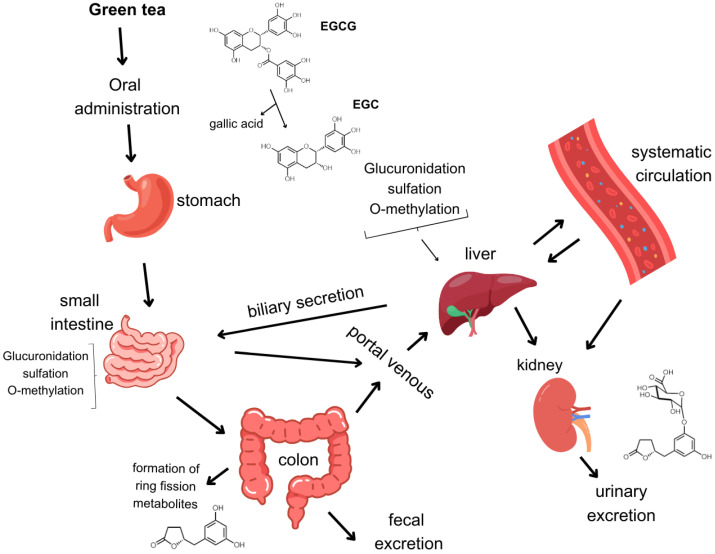
Schematic diagram of EGCG catechin metabolism in the human body.

Previous studies have also examined pathways associated with the metabolism of catechins in the liver, kidney, and gastrointestinal tract, including glucuronidation, methylation, sulfation, and ring fission (Figure 2) [20,25,26]. The route of administration significantly influences EGCG bioavailability [16]. For instance, intravenously administrated EGCG can reach all tissues in a free state (without conjugate) when compared to intragastrical and oral administration, when EGCG bioavailability ranges from 0.1 to 0.3% in rats and humans [18,27,28], while the ring fission valerolactone’s bioavailability can reach 40% [6,29]. Additionally, when discussing the metabolic pathway of EGCG, differences in the composition of intestinal microbiota can substantially impact the variability of metabolites as well as the absorption rate in humans [6,30,31]. Bacteria species such as *Eubacterium*, *Flavonifractor*, *Eggerthela*, and *Adlercreutzia* have been reported for their capacity to metabolize green tea catechins (GTCs) [32,33]. A recent study conducted by Su et al. described the metabolism of green tea catechins by human fecal microbiota, showing that a GTC concentration of 0.1 mg/mL for 48 h significantly increased the abundance of *Unidentified_Ruminococcus*, *Eubacterium*, *Enterococcus*, *Clostridium*, and *Flavonifractor* when compared to the GTC sample at 0 h [34].

## 4. Catechin Ring Fission Metabolites and Their Biological Activity

**Figure 3:**
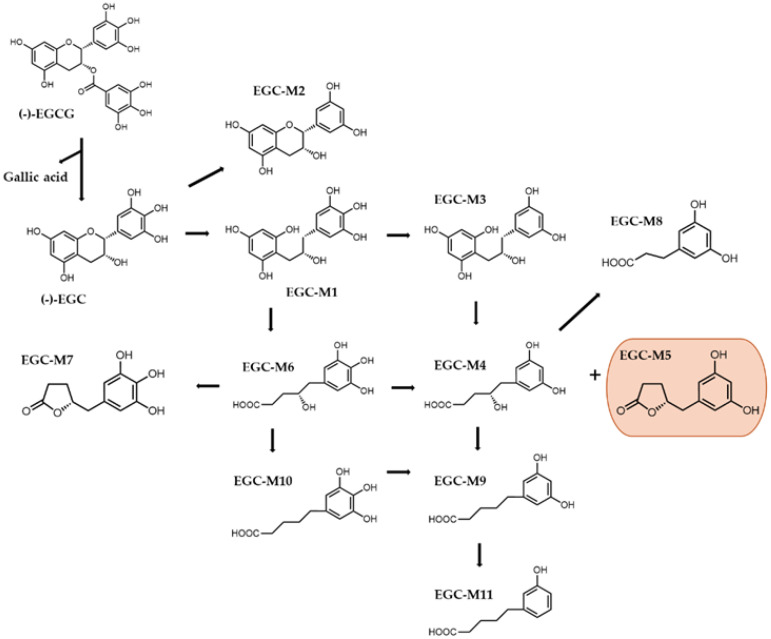
Chemical structures of EGCG metabolites derived from data provided by Takagaki et al. EGC-M5 is described as the major metabolite produced [37].

After consumption, EGCG is hydrolyzed by bacteria from the intestinal microbiota, such as *Enterobacter aerogenes*, *Raoultella planticola*, *Bifidobacterium longum*, and *Klebsiella pneumoniae*, producing EGC and GA (gallic acid) [4,35]. Upon reaching the large intestine, 11 ring fission metabolites have been described as products of EGC microbial metabolization (Figure 3). Described by Takagaki et al. in 2010, these metabolites are 1-(3,4,5-trihydroxyphenyl), 3-(2,4,6-trihydroxyphenyl)-propan-2-ol (EGC-M1), 4-dehydroxylated epigallocatechin (EGC-M2), 1-(3,5-dihydroxyphenyl)-3-(2,4,6-trihydroxyphenyl)-propan-2-ol (EGC-M3), 4-hydroxy-5-(3,5-dihydroxyphenyl) valeric acid (EGC-M4), 5-(3,5-dihydroxyphenyl)-valerolactone (EGC-M5), 4-hydroxy-5-(3,4,5-trihydroxyphenyl) valeric acid (EGC-M6), 5-(3,4,5-trihydroxyphenyl)-valerolactone (EGC-M7), 3-(3,5-dihydroxyphenyl) propionic acid (EGC-M8), 5-(3,5-dihydroxyphenyl) valeric acid (EGC-M9), 5-(3,4,5-trihydroxyphenyl) valeric acid (EGC-M10), and 5-(3-hydroxyphenyl) valeric acid (EGC-M11) [4]. Between these, EGC-M5 and EGC-M7 have been shown to be the primary metabolites in the plasma, urine, and bile of rats, mice, and humans [7].

A study conducted by Khori et al. demonstrated the metabolic fate of [4-^3^H] EGCG in rats after oral administration [36]. Following the intragastric gavage of [4-^3^H] EGCG (4 mg, 7.4 MBq/kg) to male Wistar rats, the absorption, distribution, and excretion of EGCG and its metabolites in blood, tissues, urine, and feces were assessed by tracking radioactivity using HPLC analysis. According to the results, the radioactivity of EGCG had completely disappeared in the stomach after 72 h. The highest levels of radioactivity were detected in the small intestine, cecum, and large intestine at 4 h (40.5% of the administered dose), 8 h (46.4% of the administered dose), and 24 h (13.2% of the administered dose), respectively. The reduction was substantial within 24 h and almost completely disappeared by 72 h in these tissues. The blood’s radioactivity level was initially low after 4 h, then steadily increased after 8 h, reached its highest point at 24 h, and subsequently declined. The urinary levels of EGC-M5 3-O-β-glucuronide and EGC-M5 were 68% and 16.8% of the radioactivity, respectively, after 48 h of administration. The authors suggested that orally administered EGCG is absorbed in the intestinal tract within a few hours (less than 8 h). And the absorption of EGCG metabolites and conjugates takes place in the large intestine (between 8 and 48 h). These substances are then distributed to various tissues through the bloodstream and eventually excreted in the urine [36]. In another study, Meng et al. found that the levels of EGC-M7 and EGC-M5 in mouse urine ranged from 1.5 to 8.3 μM and 3.1 to 26.5 μM, respectively, following the administration of 0.6% green tea [15]. These results provide evidence of better absorption of the metabolites compared to the intact catechins.

The primary urinary metabolites observed in healthy male volunteers after consuming 200 mL of reconstituted green tea (derived from 3 g of tea solids) were ring fission metabolites of tea catechins, including 5-(3,4-dihydroxyphenyl)-γ-valerolactone, EGC-M5, and EGC-M7, as well as their glucuronide and sulfate conjugates. These metabolites were most abundant in the urine between 12 and 24 h after tea ingestion [29]. After drinking 20 mg/kg of decaffeinated green tea, two metabolites called EGC-M7 and 5-(3,4-dihydroxyphenyl)-γ-valerolactone were detected in urine (4–8 μM) and plasma (0.1–0.2 μM) after 13 h [29]. In addition, these microbial ring fission metabolites’ combined urine excretion was 8–25 times more than that of ECG and EC [30].

Catechin metabolites have demonstrated multiple biological activities, such as anti-oxidative, anti-inflammatory and anti-diabetic effects, as well as immunomodulatory and blood pressure-lowering activities. An interesting study conducted by Takagaki et al. investigated the anti-diabetic effects of these metabolites. The capacity of EGCG metabolites to modulate glucose uptake was assessed using 2-deoxyglucose in differentiated rat L6 myoblast cells. Administration of EGC-M5, EGC-M6, EGC-M7, and EGC-M11 at a concentration of 3 μM for 15 min resulted in a significant increase in glucose absorption. The enhancement percentages were 164.2%, 165.2%, 167.6%, and 146.3% for EGC-M5, EGC-M6, EGC-M7, and EGC-M11, respectively, compared to the control cells. In addition, the study found that 30 min after oral glucose loading, the group that received EGC-M5 at a dosage of 32 mg/kg of body weight showed a substantial reduction in postprandial hyperglycemia compared to the control group that received saline. The average blood glucose level was 108.5 ± 17.2 mg/dL in the EGC-M5 group and 150.5 ± 13.6 mg/dL in the control group. This effect was also observed 15 min after oral glucose loading [37]. Takagaki et al. conducted a study using spontaneously hypertensive rats to explore the effects of a single oral dosage of the EGCG metabolites EGC-M5 and EGC-M7 on systolic blood pressure (SBP). There was a significant decrease in systolic blood pressure (SBP) two hours after administering EGC-M7 at a dose of 150 mg/kg and four hours after administering EGC-M5 at a dose of 200 mg/kg, compared to the control group [38]. Research indicates that the ring fission metabolites of catechins produced by the microorganisms in the intestines have a significant role in safeguarding against different illnesses.

### 4.1. Antioxidant Properties of Catechins and Metabolites

Catechins have potent antioxidant activity due to the number and location of hydroxyls on other chemical groups, which provides their free-radical scavenging activities [39]. Antioxidant activity is one of the most well-documented mechanisms of action of phenolic compounds, which protects DNA from damage caused by reactive oxygen species (ROS) [40]. Increasing the activity of antioxidant enzymes such as catalase, superoxide dismutase, and glutathione peroxidase that eliminate ROS directly and preventing the formation of hydroxyl free radicals are some of the mechanisms by which catechins from green tea exert this function [41]. Other pathways related to oxidative stress have also been described to be regulated by catechins [3,39]. Catechins have been demonstrated to inhibit the activation of the MAPK/AP-1 (mitogen-activated protein kinase) pathway [42,43], enhance the activation of the Keap1/Nrf2/ARE signal pathway [44,45], and block the activation of nuclear factor-κB (NF-κB) pathways [44,46,47], reactions that work together to minimize oxidative stress.

Although information about catechin metabolites’ physiological activity is still limited, the antioxidant activity of these metabolites was investigated by Takagaki and Nanjo [48]. Through the 2,2′-azino bis(3-ethylbenzothiazoline-6-sulfonic acid) (ABTS) method, they demonstrated that all ring fission metabolites including EGC-M5 had antioxidant properties at least equivalent to Trolox, although their activities were one-seventh to two-thirds of EGCG. It was found that the ring fission metabolites produced from EC or ECG, 5-(3,4-dihydroxyphenyl)-γ-valerolactone, and 5-(3-hydroxyphenyl)-γ-valerolactone, as well as EGCG metabolites such as EGC-M4, EGC-M5, EGC-M9, EGC-M10, and EGC-M11, had stronger radical scavenging abilities than parental catechins [48]. Nevertheless, taking into account the high bioavailability of these metabolites, in comparison to the low bioavailability of EGCG, it is possible that they are the main contributors to health after the consumption of green tea [48].

### 4.2. AntiCancer Properties of Catechins and Metabolites

Green tea has been reported to play an important role in cancer chemoprevention, with various mechanisms of action already being demonstrated in experimental studies [3,49]. Induction of apoptosis and cell cycle arrest, inhibition of angiogenesis, oxidative stress mitigation, and inhibition of pro-inflammatory mediators are a few of the mechanisms through which GTCs act against cancer [3,50,51]. EGCG has been demonstrated to have chemopreventive effects in the carcinogenic process by suppressing many phases of carcinogenesis, including initiation, promotion, and progression [51]. The hallmarks of cancer are characterized by several key features, including continuous cell division, ability to evade immune destruction, resistance to cell death, ability to replicate indefinitely, stimulation of blood vessel growth (angiogenesis), activation of invasion and metastasis, alteration of energy metabolism, and ability to evade mechanisms that typically restrict cell proliferation [52]. EGCG has been demonstrated to target multiple cancer hallmarks in various types of cancer [50]. For instance, in terms of cell death resistance, EGCG has been linked to apoptosis induction in breast [53], gastric [54], liver [55], and other cancers [50]. In terms of angiogenesis, EGCG has been demonstrated to inhibit tumor-secreted factors such as vascular endothelial growth factor (VEGF) in endometrial [56], lung [57], gastric [58], and breast cancer [57,59]. Other cancer hallmarks, such as replicative immortality [60,61], tissue invasion and metastasis [62,63,64], and immune system evasion [65,66,67], have also been shown to be targeted by EGCG.

Our previous study elucidated the effects of GTE (green tea extract) on two breast tumor cells, MDA-MB-231 (p53 mutant) and MCF-7 (wild-type p53), and MCF-10A (non-tumoral breast cell). Cells were exposed to different concentrations of GTE (31.2–1250 µg/mL) for 24–48 h, and cell viability was assessed in the presence and absence of pifithrin-α, a p53 inhibitor. The results showed that GTE reduces the cell viability of breast cancer cells, while having no cytotoxic effect on non-tumor cells. In the presence of the p53 inhibitor, both tumor lines treated with GTE showed increased survival levels, suggesting the involvement of p53 in GTE’s cytotoxicity. Cell migration using a wound healing assay showed that GTE reduced the migration of MDA-MB-231 and MCF-7 by 50 and 30%, respectively. The findings shed light on GTE’s mechanism of action in terms of its anticarcinogenic potential in breast cancer cells [68].

Our group conducted another investigation examining GTE’s impact and isolated EGCG on an MCF-7 three-dimensional culture. This approach was chosen to more accurately replicate the spatial arrangement of tumors in vivo, as 2D cultures have some limitations. The spheroids were subjected to GTE concentrations ranging from 162 to 2592 µg/mL and EGCG concentrations ranging from 7.8 to 62.5 µg/mL, which corresponded to the range of EGCG present in the GTE doses used. The results showed that GTE exposure starting from 648 µg/mL inhibited the formation of breast cancer spheroids and decreased cell migration more effectively than EGCG. It is important to note that the concentrations that proved toxic to the tumor cell had no cytotoxic effects on non-tumoral MCF-10A spheroids, suggesting a selectivity in the mechanism of action [69].

An additional molecular mechanism that has been proposed to explain the anticancer properties of GT catechins is the modulation of microRNA (miR) expression, downregulating the expression of oncogenic miRs and upregulating tumor-suppressive ones [70]. Oncogenic miRs such as miR-21, miR-25, and miR-27a have been reported to be downregulated by EGCG in MCF-7 (breast cancer), MM1.s (multiple myeloma), and 22Rv1 xenograft (prostate tumor) [71,72,73], resulting in decreased expression of vascular endothelial growth factor (VEGF) and IL-6 and increased expression of p53, p21, and caspase-3. Tumor-suppressive miRs such as miR-16, 34a, 145, and 200c have also been reported to be upregulated by EGCG on HepG2 (hepatocellular carcinoma) [74], HCT116 cells (colorectal cancer) [75], and different malignant neuroblastoma cell lines (SK-N-BE2, IMR-32, SH-SY5Y, and SK-N-DZ) [76,77]. The regulation of these key miRs is linked to the decrease in Bcl-2 and NF-κB and an increase in caspase-3 and PTEN, leading to cell cycle arrest, apoptosis, and decreased cell growth, invasion, and metastasis [70].

The metabolites have also been reported to have anticancer properties [78,79]. Lambert et al. described the growth inhibition effects of EGC-M7 on human colon adenocarcinoma cells (HT-29 and HCT-116) and esophageal squamous carcinoma cells (KYSE150), with an IC_50_ value ranging from 15 to 73 µM [78]. A study conducted by Hara-Terawaki et al. also investigated the inhibitory effects of the catechin metabolites produced by intestinal bacteria on HeLa cells (human cervical cancer). Eleven types of metabolites produced from EGC (EGC-M1 to EGC-M11) and four metabolites from EC (1-(3,4-dihydroxyphenyl)-3-(2,4,6trihydroxyphenyl)propan-2-ol (EC-M1), 4-hydroxy-5-(3,4dihydroxyphenyl)valeric acid (EC-M3), 5-(3,4-dihydroxyphenyl)levulinic Acid (EC-M7), 5-(3,4-dihydroxyphenyl)valeric Acid (EC-M9)) were incubated with HeLa cells with concentrations ranging from 0.4 to 50 µg/mL for 72 h, and proliferation was assessed using an MTT assay. Among the metabolites tested, EGC-M2, EGC-M7, EGC-M9, and 5-(3,4-dihydroxyphenyl)valeric acid inhibited the proliferation of HeLa cells, demonstrating the metabolites’ potential and biological efficacy against cancer [79].

## 5. Modulation of Tumor Microenvironment by Green Tea Catechins and Major Metabolites

Extensive evidence shows that solid tumors are composed not only of cancerous cells but also of substantial alterations in the surrounding tissue, known as the tumor microenvironment (TME). The TME is recognized as a crucial factor in tumor development and is a potential target for therapeutic interventions [80,81,82,83]. The tumor microenvironment consists of diverse cell types, including tumor cells, leukocytes, adipocytes, fibroblasts, and endothelial cells. Soluble factors such as cytokines and growth factors are also part of this microenvironment, which can alter critical physical properties such as pH and oxygen content [80,82]. The secretion of growth factors such as TGF-β and VEGF and cytokines such as CCL2 and IL-10 by tumor cells enables angiogenesis and recruitment of tumor-associated macrophages, promoting tumor growth and metastasis [82,84].

Fibroblasts are the most abundant cells in the tumor microenvironment, also called tumor-associated fibroblasts (TAFs) [85]. They are known to secrete a variety of soluble factors, such as metalloproteinases (MMPs), enzymes that are related to changes in the extracellular matrix, promoting tumor invasion [82]. Remodeling of the extracellular matrix, influenced by the expression of MMPs, is a critical step in the metastatic process of cancer and has been established as a challenge for both prognosis and treatment.

**Figure 4:**
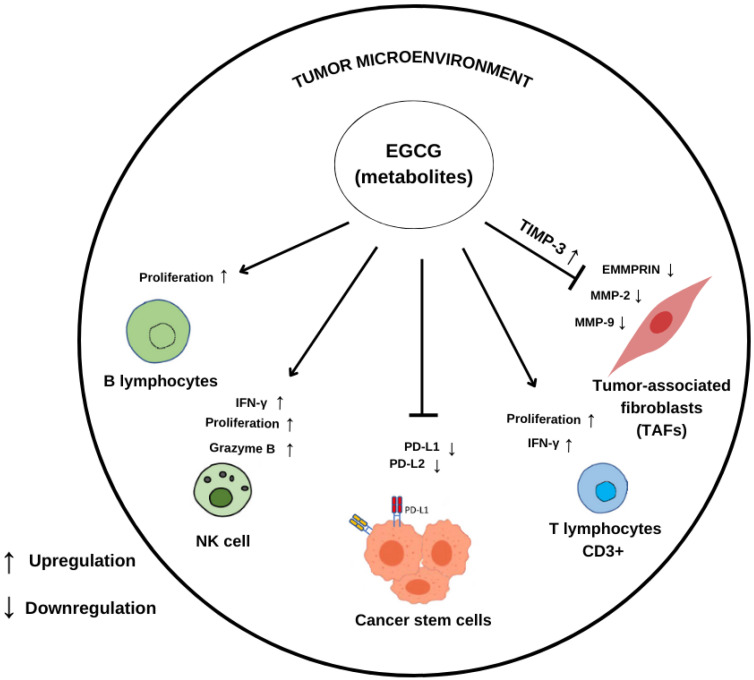
Schematic representation of green tea catechins’ and metabolites’ potential effects on immune cells in the tumor microenvironment.

The effects of EGCG and GTP (green tea powder—commercial product) on these markers have been studied (Figure 4). In MCF-7 and MDA-MB-231 cancer cell lines, the induction of the tissue inhibitor of metalloproteinases-3 (TIMP-3) mRNA was associated with EGCG and GTP. One of the main mechanisms by which GTP inhibits the expression of MMPs may be the epigenetic induction of TIMP-3, which downregulates MMPs [86].

Farabegoli et al. showed EGCG’s ability to downregulate EGFR (epidermal growth factor receptor), MMP-2, MMP-9, and EMMPRIN (extracellular matrix metalloproteinase inducer), which is a glycoprotein able to activate MMPs in tamoxifen-resistant breast cancer cell line (MCF-7Tam). MCF-7Tam and parental MCF-7 cells were treated with EGCG at concentrations ranging from 10 to 100 µg/mL. It was found that a concentration of 50 µg/mL considerably reduced the levels of the components being studied. Phosphorylation of EGFR at Tyr-992, Tyr-1045, and Tyr-1068 was elevated in MCF-7Tam compared to MCF-7, and it was likewise diminished by EGCG therapy. Research findings indicate that EGCG has the ability to reduce the tamoxifen-resistant characteristics of MCF-7Tam cells, hence reducing their invasion [64].

Tumor-infiltrating lymphocytes have emerged as a major participant in the tumor microenvironment. Most of the infiltrating lymphocytes is composed of T lymphocytes, which can be divided into CD4^+^ T helper cells (Th1, Th2, and Th17), CD4^+^ Treg, CD8^+^ T cells, and natural killer cells [81,87]. Elevated levels of Treg are related to a worse prognosis, as they suppress immune responses to tumor antigens [87].

One of the hallmarks of cancer cells is tumor immune evasion, which can be promoted by several mechanisms, including the production of inhibitory ligands on the cell surface such as programmed cell death ligand 1 (PD-L1). T cell exhaustion is caused by PD-L1′s interaction with the programmed cell death 1 (PD-1) receptor on T cells [9]. Monoclonal antibody treatment reactivates T cell-mediated tumor killing by blocking this inhibitory connection. Therefore, great clinical success has been achieved with PD-1/PD-L1-based cancer immunotherapy in the treatment of advanced malignancies, including melanoma [88], non-small cell lung cancer [89,90], and others [91,92].

To investigate the effects of the major catechin in tea, EGCG, on the PD-1/PD-L1 immune checkpoint, Menon et al. conducted a study that investigated the effects of EGCG using both in vitro and in vivo experiments. Three human metastatic melanoma cell lines (1205 Lu, A375, HS294 T) were treated with either 10 µM of EGCG, 10 ng/mL IFN-γ, or a combination of both. IFN-γ has been identified for its ability to stimulate the production of PD-L1/L2. Consequently, the administration of IFN-γ resulted in an increase in the expression of PD-L1 and PD-L2 on the cell surface of human melanoma cells. When EGCG was used together with IFN-γ, the increase in PD-L1/L2 caused by IFN-γ was inhibited in all three evaluated cell lines [65]. The in vivo investigations conducted on C57BL/6 mice, using doses of 50 or 100 mg/kg, revealed that the tumor-inhibitory effect of EGCG was facilitated by CD8^+^ T cells and was equivalent to the effects of anti-PD-1 treatment. Nevertheless, their methods of operation were distinct. Unlike anti-PD-1 therapy, which hinders the interaction between PD-1 and PD-L1, EGCG inhibits JAK/STAT signaling and reduces PD-L1 expression in tumor cells, leading to the reactivation of T cells. To summarize, the research indicates that EGCG enhances the body’s immune response to tumors in melanoma by reducing the activity of JAK-STAT signaling. EGCG could potentially be utilized as an alternative therapeutic approach to target the PD-L1/PD-L2-PD-1 axis, specifically in malignancies [65].

Rawangkan et al. described EGCG as an alternative inhibitor of the PD-1/PD-L1 checkpoint, inhibiting tumor expression of PD-L1 in lung cancer, in vitro, and animal models, via inhibition of IFN-γ and EGF. Non-small-cell lung cancer (NSCLC) cell lines (A549 and Lu99) were exposed to different concentrations of EGCG. In A549 cells treated with 50 µM EGCG and 100 µg/mL GTE, IFN-γ-induced PD-L1 was reduced by 40 to 80%. Likewise, the Lu99 cells showed a reduction of 37–50% in EGF-induced PD-L1 expression when pre-treated with 50 µM EGCG. In experiments in vivo, female A/J mice with induced lung carcinogenesis by 4-(methylnitrosamino)-1-(3-pyridyl)-1-butanone (NNK) received oral administration of 0.3% GTE in drinking water. This treatment resulted in a reduction of 70% of PD-L1 positive cells, when compared to the NNK group. In addition, co-culture experiments using F10-OVA melanoma cells and tumor-specific CD3^+^ T cells showed that EGCG reduced PD-L1 mRNA expression by about 30%, while also restoring interleukin-2 mRNA expression in the CD3^+^ T cells. These findings suggest that EGCG acts like an immune checkpoint inhibitor and can partially restore the cellular activity of T lymphocytes, reducing tumor growth [66]. The results serve as evidence of catechins’ potential to modulate immune checkpoints, which are often targeted in advanced cancer immunotherapies.

In a murine leukemia mouse model, Huang et al. described that oral treatment with or without EGCG at 5, 20, and 40 mg/kg for two weeks increased the percentage of CD3^+^ cells (T-lymphocytes) and CD19^+^ cells (B-lymphocytes). EGCG boosted T cell proliferation at 40 mg/kg but increased B cell proliferation at all three dosages. It also raised natural killer cell activity at 5 mg/kg and the phagocytosis of macrophages at 40 mg/kg [93].

Lowe et al. demonstrated higher leukocyte activation in healthy people when supplemented with 300 mg of GTE for 14 days. Although the leukocyte count was not influenced by supplementation, there was an increased secretion of myeloperoxidase and lactoferrin, molecules that activate mature neutrophils and monocytes [94]. Another study carried out with isolated neutrophils showed greater production of superoxide and myeloperoxidase by these cells when exposed to a concentration of 10 µL of green tea extract, when compared to control [95].

A study conducted by Kim et al. demonstrated the immunoregulatory effects of EGC and EGC-M5 on CD4^+^ cells by measuring ATP levels. Spleens were collected from 8−10-week-old BALB/c mice, and CD4^+^ splenocytes were isolated. Thereafter, CD4^+^ T cells were exposed to various green tea catechins, including EC, (−)-catechin (C), EGCG, (−)-gallocatechin-3-O-gallate (GCG), ECG, (−)-catechin-3-O-gallate (CG), (−)-gallocatechin (GC), and EGC. Additionally, 11 different types of green tea catechin metabolites (EGC-M1–EGC-M11) and phytohemagglutinin (PHA) were used as a positive control. Consequently, the metabolites lacking the hydroxy group on the B ring of the flavan structure, including EGC-M2, EGC-M3, EGC-M4, EGC-M5, EGC-M8, EGC-M9, and EGC-M11, stimulated enhanced CD4^+^ T cell activity. The data also indicated that 5-(3′,5′-dihydroxyphenyl)-γ-valerolactone-3′-O-glucuronide (EGC-M5-glucuronide) exhibited a considerable increase in CD4^+^ cell activity, similar to EGC-M5 [96].

Given that EGC-M5 is the primary metabolite found in both human and rat urine and has been shown to have superior absorption, more studies were conducted utilizing EGC-M5. IFN-γ and IL-2 are recognized as T cell stimulants and agents that promote the function of activated CD4^+^ T cells. Additionally, IFN-γ can inhibit the growth of cancer cells by inducing NK cells to exhibit cytotoxic activity, thereby promoting the differentiation of activated CD4^+^ T cells into type 1 T helper (Th1) cells. An essential part of NK cells’ anticancer immunological activity is determined by their IFN-γ level. To investigate this pathway, splenocytes were incubated in the presence or absence of EGC-M5 or EGC (0, 5, 10, 25, and 50 μM) for 72 h. Subsequently, the levels of IFN-γ and IL-2 were measured. EGC-M5 did not have any significant impact on the level of IL-2. However, the production of IFN-γ increased significantly in splenocytes treated with 10, 25, and 50 μM EGC-M5 compared to those treated with the same dosages of EGC [96]. Table 1 provides summarized information on the modulatory effects of green tea catechins and major metabolite on the tumor microenvironment.

In the aforementioned study, YAC-1 murine lymphoma cells were co-cultured with NK cells obtained from BALB/c mice. These mice were given a daily oral dose of 200 µL of EGC-M5 and EGC solution at a concentration of 10 mg/kg per body weight for a duration of 14 days. The cytotoxic activity of NK cells against YAC-1 cells significantly increased compared to the EGC intake and control groups, resulting in enhanced cell death in YAC-1 cells. EGC-M5 is hypothesized to suppress the growth of YAC-1 cells by enhancing the activity of CD4^+^ T cells and the cytotoxicity of NK cells. This, consequently, leads to an increase in the production of IFN-γ and the proliferation of granzyme B^+^ NK cells [96].

Natural killer (NK) cells generate perforin and granzyme B, which induce apoptosis and necrosis in target cells. These enzymes form perforations in the membrane of the targeted cell, thereby enabling its demise. Flow cytometry was used to evaluate granzyme B^+^ NK cells and perforin^+^ NK cells in spleens from mice fed with EGC-M5. The administration of EGC-M5 resulted in an increase in granzyme B^+^ NK cells but did not affect the populations of perforin^+^ NK cells [96].

Green tea catechin metabolites were suggested to induce immunological activation through NK cell cytotoxicity and CD4^+^ T cell activity. More precisely, it was found that EGCG metabolites without a 4′-hydroxyphenyl group enhanced the activity of CD4^+^ T cells and that EGC-M5 exerted anticancer benefits by promoting NK cell cytotoxicity. Consequently, these discoveries are expected to have positive effects on the prevention and treatment of cancer, as well as on studies into the biological activity of intestinal metabolites in the body [96].

## 6. Conclusions

Cancer remains one of the leading causes of mortality and morbidity globally, despite significant advances in treatment. The tumor microenvironment is a promising target for therapeutic intervention, as it comprises both cancer and multiple immune cells that can paradoxically either eradicate or support tumor progression.

Although anticancer drugs are effective in treating cancer, they often have unfavorable side effects. It has been demonstrated that natural products can significantly contribute to both the prevention and suppression of cancer by modulating various biological processes, which may help to reduce side effects that limit treatment.

In this review, we compile evidence showing that green tea catechins and their metabolites can modulate cells within the tumor microenvironment and, therefore, may serve as a potential aid in the fight against cancer. When studying the benefits of green tea catechins, in vitro and in vivo studies have limitations, and information about the biological effects of the metabolites is scarce. New experimental models are needed to better elucidate the effects of green tea and its catechins’ ring fission metabolites on cancer and the immune system, in an attempt to understand their potential impact on the tumor microenvironment.

## Figures and Tables

**Table 1:** Potential effects of green tea catechins and major metabolite on the tumor microenvironment.

Compound/ Concentration Used	Experimental Model	Modulatory Effects ↑ Upregulation ↓ Downregulation	Reference
EGCG 20 μM	Breast cancer cells MCF-7 MDA-MB-231	↑ TIMP-3 ↓ MMP-2 ↓ MMP-9	[86]
GTP 10 μg/mL	Breast cancer cells MCF-7 MDA-MB-231	↑ TIMP-3 ↓ MMP-2 ↓ MMP-9	[86]
EGCG 50 µg/mL	Tamoxifen-resistant breast cancer cell MCF-7 Tam	↓ EGFR ↓ MMP-2 ↓ MMP-9 ↓ EMMPRIN	[64]
EGCG 10 µM	Melanoma cell lines 1205 Lu, A375, HS294T	Inhibits expression of PD-L1/L2	[65]
EGCG 50 or 100 mg/kg	In vivo C57BL/6 mice	Inhibits JAK/STAT signaling ↓ PD-L1 T cell reactivation	[65]
EGCG 50 µM	NSCLC cell lines A549 and Lu99	Inhibits the PD-1/PD-L1 checkpoint ↓ EGF-induced PD-L1 expression	[66]
GTE 0.3%	In vivo A/J mice with induced lung carcinogenesis	↓ PD-L1 positive cells	[66]
EGCG 10 to 30 µM	Co-culture of Melanoma F10-OVA with CD3^+^ T cells	↓ PD-L1 ↑ Interleukin-2 ↑ T lymphocytes activity	[66]
EGCG 5, 20, and 40 mg/kg	In vivo BALB/c mice with induced leukemia	↑ B lymphocyte ↑ T lymphocyte ↑ NK cell activity ↑ Macrophages phagocytosis	[93]
GTE 300 mg/14 days	In vivo Human model	↑ Leukocyte activation ↑ Myeloperoxidase ↑ Lactoferrin	[94]
GTE 10 µL	neutrophils	↑ Superoxide ↑ Myeloperoxidase	[95]
EGC-M5 5, 10, 25, and 50 μM	CD4^+^ T cells	↑ CD4^+^ T cell activity	[96]
EGC-M5 5, 10, 25, and 50 μM	Splenocytes	↑ IFN-γ	[96]
EGC-M5 10 mg/kg	In vivo BALB/c mice	↑ granzyme B^+^ NK cells	[96]
